# A Case of Gun-shot Wound of the Femur

**Published:** 1866-07

**Authors:** J. R. Lothrop


### ART. II.—*A Case of Gun-shot Wound of the Femur.* By J. R. Lothrop, M. D.

August Ferkie of the 100th N. Y. V., was wounded in an engagement which took place August 16, 1864, at Deep Bottom, Va., on the north bank of the James River, during the siege of Richmond. After his wound he was left on the field and taken prisoner. He was wounded about 10 A. M., and the next morning was taken to Richmond. The wound was made by a minie ball, which shattered the left femur in its upper third, near the point of its union with the middle third. The ball was probably split into several pieces, as subsequently two pieces escaped from the broken thigh, and a portion inflicted a wound upon the right, though it did not perforate it. A portion of the ball perforated the left thigh, then, and lodged in the right—being, as was supposed by the surgeon who examined him, stayed in its progress by the right femur.

He remained a prisoner about a week, then was exchanged and taken to the military hospital at Annapolis. During the time he was a prisoner he had no treatment, and little food, though the rebel surgeons were liberal in examinations, using the finger as a probe, and discussed the propriety of enlarging the wound to remove the loose pieces of bone. Nothing, however, was done till he reached the hospital at Annapolis. Then the limb was suspended for a period of four weeks, and afterwards extension by weights employed and continued for some time; as nearly as he can remember about 11 weeks, a weight of 18 pounds being used. While there the portions of the ball spoken of came out of the wound. In seven months he was removed to Baltimore, where he remained till he was discharged, about one year ago. He came to Buffalo, and three months since entered the Buffalo General Hospital. While under treatment in Baltimore several pieces of bone came from the limb, one two inches in length, the others small. He was able to walk about on crutches in eleven months after he was wounded. Up to the present time there has been a discharge from the wound, and pain more or less constant. At various times recently, small pieces of bone, exfoliations probably, have worked their way out. Still he is able to walk some portions of the day, with the aid of crutches.

When admitted to the hospital, three months since, both wounds, viz: that of entrance and exit were unhealed, and discharging pus quite freely. There were besides on the side of the wound of entrance, several openings four inches above. The sinuses leading from all these openings took the direction of the seat of fracture. The limb was shortened nearly three inches, the foot rotated inwards, and the motions of all the joints limited. At the point of union there was great irregularity. The short upper fragment was displaced upwards and outwards rather more than is usually observed in fractures high up in the femur, giving to the outside of the thigh a rounded and prominent appearance. At the seat of fracture there was a great thickness of bony casing, uniting the ends and enclosing the fragments, living and dead. With all this, though the man had been crippled and worn down by nearly two years of exhausting efforts of repair, his condition warranted an attempt to hasten recovery by an operation. The suppurative discharge indicated the presence of fragments of necrosed bone, imprisoned within the new bony casing; although no roughness could be detected by the probe.

The aim of an operation was, the removal of these fragments. For this purpose the trephine was applied to the bone, at the wound of entrance, near the point of fracture, to enlarge the opening which already existed, and through which pus found exit. A section of bone having been removed, an opening was made into a bony cavity large enough to permit an exploration. Several fragments of dead bone were found and removed, the largest being nearly an inch in length. These sequestra were below the opening made in the bone, and appeared to have had connection with the lower part of the shaft. This might be expected from the length of time which had elapsed since the original injury, viz: nearly two years. According to the statements of Mr. Curling fragments are longer in effecting their separation from the lower than from the upper portion of the shaft in cases of compound fracture of the femur. The cavity was then washed out and left to fill up by granulation; no closure of the soft parts being attempted. No great disturbance of the system followed this operation, and though the purulent discharge was for a time increased, it soon became less than before the operation, and some of the sinuses ceased to give exit to pus. At the present time, three months since the operation, there seems good prospect that the purulent discbarge will soon cease. But if such a result should not follow, it will in no way bring into question the propriety or advantage of the operation; for, the removal of sequestra by operation is a positive gain over the slow elimination by natural methods. The continuance of the discharge would rather indicate the presence of other sequestra undetected, than that new death and exfoliation had followed from the violence done to the bone by the operation.

This is a case similar to many which have been and will be reported, of recovery from comminuted fracture caused by gunshot wound. As reported many have been made to appear much better as to their results than they ought. The course in many cases seems to have been as follows: After a time there has appeared to be union, the fragments seemingly retaining their life, and becoming incorporated as sound bone in the restored femur. The external wound, has also often closed, and the cure seemed complete. The reasoning has been that the continuity of the periostium was not disturbed, and the fragments retaining their periostial connection and consequently their life, were able to make, like living bone, a definitive union with each other and with the main portion of the shaft; and thus in the end, restoring the femur not to perfect length and form, but consolidated apparently, and ensuring the saving of the limb. Some cases have so resulted, and as far as they go, furnish argument for the propriety of attempting to save a comminuted femur at whatever point broken. But others, and the number will be larger than has been supposed, after a seeming recovery, even to closing of the wounds of the soft parts have, when the use of the limb has been resumed, at a longer or shorter interval, become first painful, then inflamed and swollen, ending in abscess, purulent escape, and prolonged suppuration. The fragments closed in by new bony formation, have not retained life from the first, although they gave promise of being joined as living bone in a restored shaft; have in the end acted like foreign bodies, brought about the inevitable result of death of bone, viz: pain, inflammation and continued suppuration. In time they will escape, and use of the limb, little or much crippled, as the case may be, will be obtained; but always at a great expense of time and strength. In many cases an operative procedure like that described above is not only proper, but of great advantage. But in cases of these "cured" comminuted fractures, even with the aid we can give by removing the sequestra, we may expect long years of suffering from repeated abscesses, exhausting suppuration, exfoliation, and such depression of the vitality as to lead to phthisis; or if not, to long continued feeble health, chills, inability to endure cold; to say nothing of shortening and deformity which can hardly be avoided.

Several interesting questions arise from a review of the above case. In the first place the limb was saved. This of itself is a result, according to most military experience, to be seldom looked for. When we are made fully acquainted with the experience of our military surgeons in such cases, we may find that gun-shot comminuted fractures of the femur are much more capable of conservation than has been supposed. But military experience previous to our own, has not been very encouraging, as to any expectation of success. Though shortened, deformed, painful, and attended with necrosis and suppuration, the result is much better than can be obtained in many cases. Death is too often the termination. It is impossible to state the proportion, where attempts to save are made, in which life is saved even with an ill conditioned limb. But the per centage has not been very large, as can be readily ascertained by examining the records of military surgery at present existing. The extensive experience of the Crimean campaign furnishes but few in number, of cases saved; among the French hardly any. Reports, here and there, by different surgeons, indicate that our experience in the late war was attended with more favorable results, but no definite conclusion can be arrived at till the entire statistics are published.

This directs our attention to the propriety of attempting to save. Should amputation be at once performed? The determination of this question depends upon the site of the fracture. If in the upper third, all military surgeons, up to a late period, agree that an attempt should be made to save, for though a large number of those in which the attempt is made will die, yet amputation in that situation is probably more fatal. Amputation in the upper third has proved so fatal, that in some wars, as in that of the

Crimea, it was almost abandoned as hopeless. The proportion of deaths is nearly ninety per cent. ; so that, however unpromising the effort to save may be, it can hardly fail to be less so than immediate amputation—secondary amputation being, from the nature of the case, entirely out of the question. In the Hospital of the Invalides M. Hutin collected records of twenty-four cases of limbs saved after comminuted fracture of the femur above the middle, but found no evidence of a case of recovery after primary amputation for fracture in that situation. When the fracture is in the middle or lower third, but especially in the former, the case is just the opposite. In those situations amputation has given by far the best results. If the question were equal as to saving life, it certainly seems that a good stump to which an artificial limb can be attached, is to be preferred to a twisted and shortened limb, poor at best, and only to be saved through years of suffering. But amputation at these points affords better results in the saving of life. If the two points are compared with each other, it appears that there is a much greater certainty of saving life and limb when the fracture is in the lower, than when in the middle third, unless too near or extending into the knee-joint. Non-union, as is well known, is most usually in the middle third.

The next point suggested by the case, is that relating to removal of the fragments. It appears that the rebel surgeons were disposed to remove them, but did not. No attempt was suggested afterwards, by the surgeons at Annapolis, as the period when such a proceeding was feasible, had probably passed; as after inflammation had set in the attempt would de more likely to do harm than good. In regard to the removal of fragments, it seems as if the propriety of so doing, soon after the injury, could hardly be questioned, when they are at all loose. If wholly separated, removal is the only proper course, for their death is certain, and their elimination by long process is inevitable. But if loosely connected, so that their death is by no means certain, there is some question as to what is best. It would seem as if all doubt as to the preservation of vitality in these fragments, should weigh in favor of their removal. They become incorporated in the new bone, and if portions of them subsequently die and exfoliate, they keep up fistulous openings for a long time, and they may set up an inflammatory action which will extend the destruction to bone which would otherwise have remained healthy.

The destructive splintering and displacement of fragments which is the very common result of a fracture by a conical ball, renders the question of removal more clear. The comminution is usually extensive, and moreover the fragments may be so disturbed as to lie at various angles to the axis of the shaft; and even by being interposed prevent that apposition of the ends which favors consolidation. Fragments which have a firm connection and are not much disturbed, are properly allowed to remain, though they may subsequently exfoliate. Judging from results, had the fragments been removed in this case, as suggested by the rebel surgeons, it would have been far better than to have allowed them to remain. In regard to the method of removal, it appears from testimony, that enlargement of the wound of exit is preferable to enlarging that of entrance. The force of the ball generally drives the broken pieces and small splinters in that direction, and therefore it is easier to reach on that side the portions which ought to be removed.

Surgeons are not agreed as to extension in cases of comminuted gun-shot fracture. There is evidently danger of producing such a separation of the ends of bone, as will delay if not interfere with union. It seems to be a measure of doubtful propriety. But a good deal depends upon the extent comminuted, and upon the shortening caused by muscular contraction when not opposed by extension. If the bone is much comminuted and many fragments are removed, the shortening by muscular effort should be unopposed, and on the other hand when too great it should be counteracted. It seems in this case a weight of 18 pounds was found necessary, at least we may conclude so, since considerable shortening took place, even with the amount of extension employed. Apart from results, which may be held to determine its necessity, the weight employed seems much greater than we should suppose would be found of advantage. Probably all requisite care to avoid rotation was exercised, inasmuch as whatever the care taken to avoid it; some twisting must be expected.

We may conclude in review of the above case that, thus far, the result has been as good as we are warranted in expecting in cases of such severity. Though the limb saved is such a one as is hardly worth keeping, yet recovery even with a poor limb, is the best of which the case was capable. It is to be set down among the results which are justly entitled rare. Keeping out of view the question of saving life, a crooked, twisted and shortened limb is a little better than a stump so short as to preclude the idea of using an artificial limb. But taking into account, that recoveries after amputation in the upper third are extremely exceptional, we cannot but conclude that it was better to adopt a course in which recovery is rare, rather than one in which it is exceptional. In this case the result is more surprising when we consider the unfavorable circumstances in which the patient was placed soon after his wound, viz: a week's captivity, during which very little attention could or was likely to be given him, and several removals within the first ten days. That the attempt was made to save rather than amputate, may have been mere chance, for we are not yet informed what was the practice in such cases in the rebel army, but there seems to have been no intention of resorting to the latter, as far as can be ascertained from the patient's statements. It is probable that they did not think amputation advisable.

